# The impact of *Clonorchis sinensis* infection on immune response in mice with type II collagen-induced arthritis

**DOI:** 10.1186/s12865-020-0336-6

**Published:** 2020-02-17

**Authors:** Xiangyang Li, Ying Yang, Suping Qin, Fanyun Kong, Chao Yan, Wanpeng Cheng, Wei Pan, Qian Yu, Hui Hua, Kuiyang Zheng, Renxian Tang

**Affiliations:** Jiangsu Key Laboratory of Immunity and Metabolism, Department of Pathogenic Biology and Immunology, Xuzhou Medical University, Xuzhou, 221004 Jiangsu Province, People’s Republic of China

**Keywords:** *Clonorchis sinensis*, Rheumatoid arthritis, Immune response

## Abstract

**Background:**

*Clonorchis sinensis* infection could trigger strong immune responses in mice and humans. However, whether the *C.sinensis* infection has an impact on arthritis is unknown. Here we investigated the effect of *C.sinensis* infection on type II collagen-induced arthritis in BALB/c mice.

**Results:**

The mice were firstly infected with 45 *C.sinensis* metacercariae by oral gavage. Four weeks later, arthritis in mice was induced by type II collagen. Joint inflammation with severe redness and swelling in hind paws was observed in type II collagen-induced arthritis (CIA) mice. Besides, the physical activity was significantly reduced, but the respiratory exchange ratio was increased in CIA mice. Compared with CIA mice, *C.sinensis* infection could increase the severity of arthritis in CIA mice, based on the results of disease score and pathological changes. Compared to CIA mice, increased neutrophils and Ly6C^hi^ monocytes, decreased B cells and CD4^+^T cells, were found in *C.sinensis* infected CIA mice. Besides these, *C.sinensis* infected mice also displayed significantly higher levels of serum IL-4 and IL-17 than those in CIA mice.

**Conclusions:**

Taken together, our data suggest that *C.sinensis* infection have a bad effect on arthritis, and could induce the abnormality of the immune response in mice with CIA.

## Background

Rheumatoid arthritis (RA) is a kind of autoimmune disease, which is characterized by chronic joint inflammation, synovial membranes proliferation, and vasculogenesis, and could lead to cartilage and bone destruction [1]. It is estimated that 24.5 million people are affected by RA [2], and the published reports have been shown that the immune responses mediated by neutrophils, T cells, B cells, macrophages and associated cytokines, including TNF-α, interleukin (IL)-6, IL-17 and IL-1, play critical roles in RA [3]. Therefore, further understanding the factors that could affect the immune response in RA maybe help us find potential strategies for the treatment of the disease.

It is well known that parasitic infection is capable of inducing the host immune response towards a strong type 2 immune response, which is could be induced by the activation of Th2 cells with the secretion of IL-4, IL-5 and IL-13 [4, 5]. Besides these, T helper 17 (Th17) cells, Th9 and T regulatory (Treg) cells also participate in the immune response during parasitic infection [6]. Furthermore, current studies suggest that early infection of certain parasites may prevent the development of autoimmune diseases, including allergic disease and RA [7], and the mechanisms are associated with immune responses mediated by parasite infection. For example, it has been shown that *Schistosoma japonicum* infection and *Trichinella spiralis* derived antigen could reduce the severity of collagen-induced arthritis [8], via immune reactions related to IL-4 and Treg Cells mediated by parasite antigens in CIA mice.

Clonorchiasis, induced by *Clonorchis sinensis*, is widely prevalent in the Asian region, including China, Korea, Vietnam and the far east regions of Russia. Just like other helminths, *Clonorchis sinensis* infection could stimulate immune response characterized by Th2-dominated response and the imbalance of Treg/Th17 [9, 10]. Until now, the effect of *Clonorchis sinensis* infection on arthritis has not been reported. The main aim of the current study was to assess the effect of *Clonorchis sinensis* infection on the immune response on collagen-induced arthritis in mice.

## Results

### Mice infected with *C.sinensis* metacercariae

**Fig. 1:**
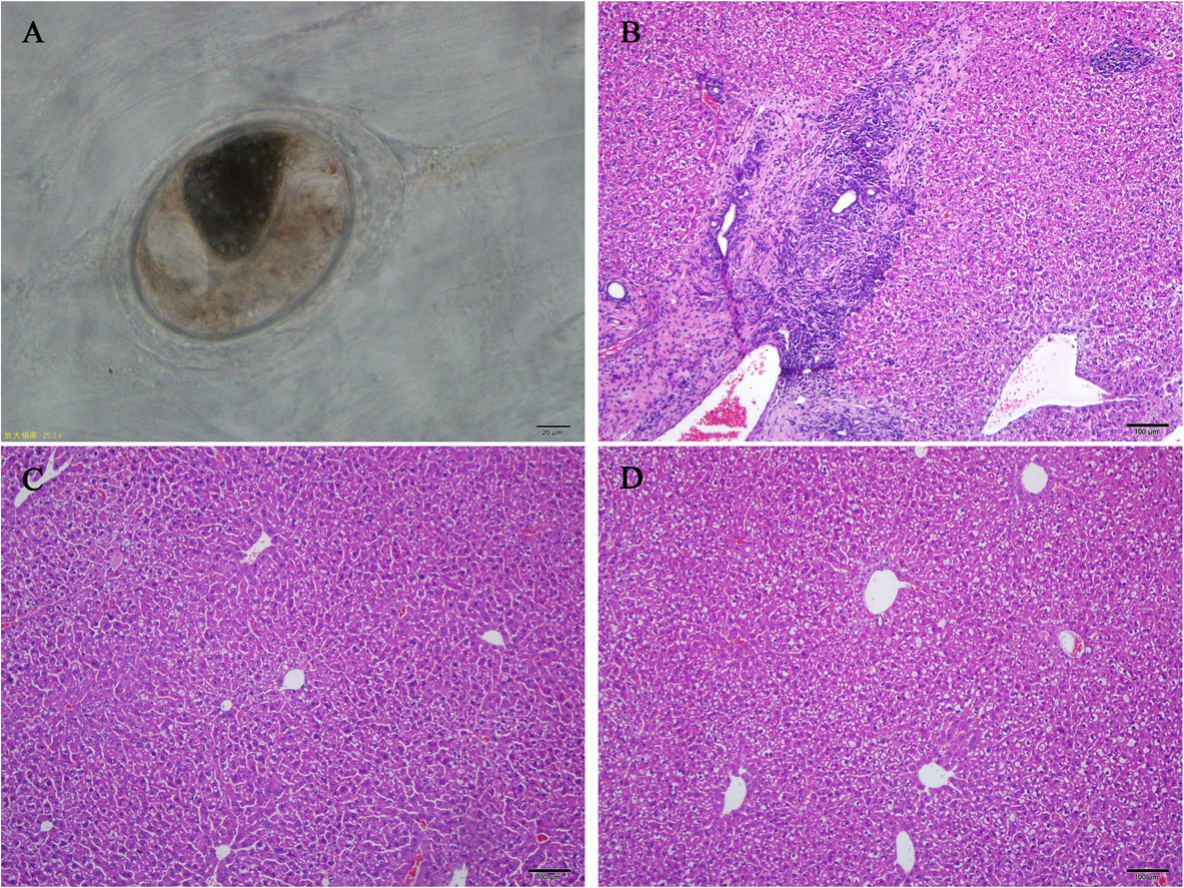
Mice infected with *C.sinensis* metacercariae. **a** *C.sinensis* metacercariae were collected from fish and examination by microscope. **b** *C.sinensis* + CIA group. Histological of liver tissues from *C. sinensis*-infected mice stained with hematoxylin and eosin (H&E). Histological structure of liver was destroyed. Massive inflammatory cells infiltrated around the portal area accompanied by collagen hyperplasia and cholangiocyte proliferation. **c**, **d** CIA group and Control group. Histological of liver tissues from the CIA and control group mice stained with hematoxylin and eosin (H&E). In both the CIA and control group, the histological structure of liver in mice was normal, without inflammatory cell infiltration and collagen hyperplasia

As shown in Fig. 1, the CIA mice were successfully infected with *C.sinensis*, and the parasite infection could induce the pathological damage of the liver in CIA mice. In portal areas of the liver of the *C.sinensis* infected CIA mice, accompanied with hepatic fibrosis, the extensive inflammatory cell infiltrations were observed.

### *C.sinensis* infection increases the joint swelling and clinical score

**Fig. 2:**
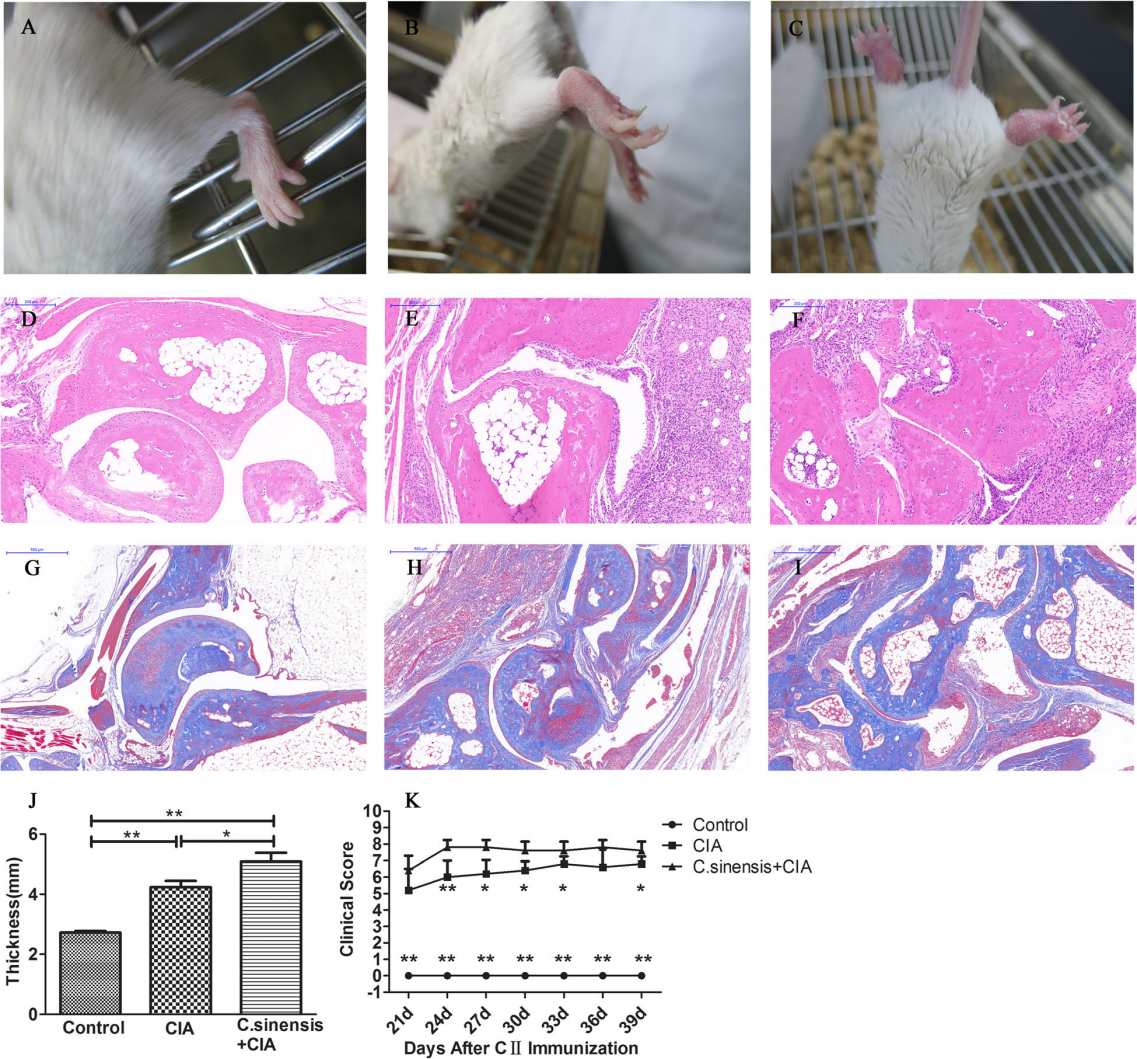
Effects of *C.sinensis* infection on the development of CIA in BALB/c Mice. **a-c** Representative images of arthritic limbs at day 39. **a** normal control mice, (**b**) CIA mice, (**c**) CIA and *C.sinensis* infection mice showing severe redness and swelling in both hind paws. **d-f** Images are H&E staining of ankle joints of mice. **d** normal control mice, (**e**) CIA mice, (**f**) CIA and C.sinensis infection mice showing inflammatory infiltration, synovial hyperplasia,pannus formation and the destruction of joints. **g-i** Images are masson staing of ankle joints of mice. **g** Normal control mice, (**h**) CIA mice, (**i**) CIA and *C.sinensis* infection mice showing the proliferation of collagen fibers and cartilage destruction. **j-k** Data show the thickness of ankle joints and the total score of four limbs after CII immunization (*n* = 5/group). Asterisks mark statistically significant difference (*P < 0.05,**P < 0.01)

Around 4 weeks after type-II collagen immunization, the mice, in the CIA group and *C.sinensis* + CIA group, developed the signs of arthritis. As the results showed in Fig. 2, the paws and knees of *C.sinensis* infected arthritic mice were more swollen than these mice in the CIA group. Moreover, the clinical scores of *C.sinensis* infected arthritic mice on day 24, 27, 30, 33 and 39, were significantly higher than the CIA group (Fig. 2).

### The changes in ambulatory activity and indirect calorimetry after C.sinensis infection in CIA mice

**Fig. 3:**
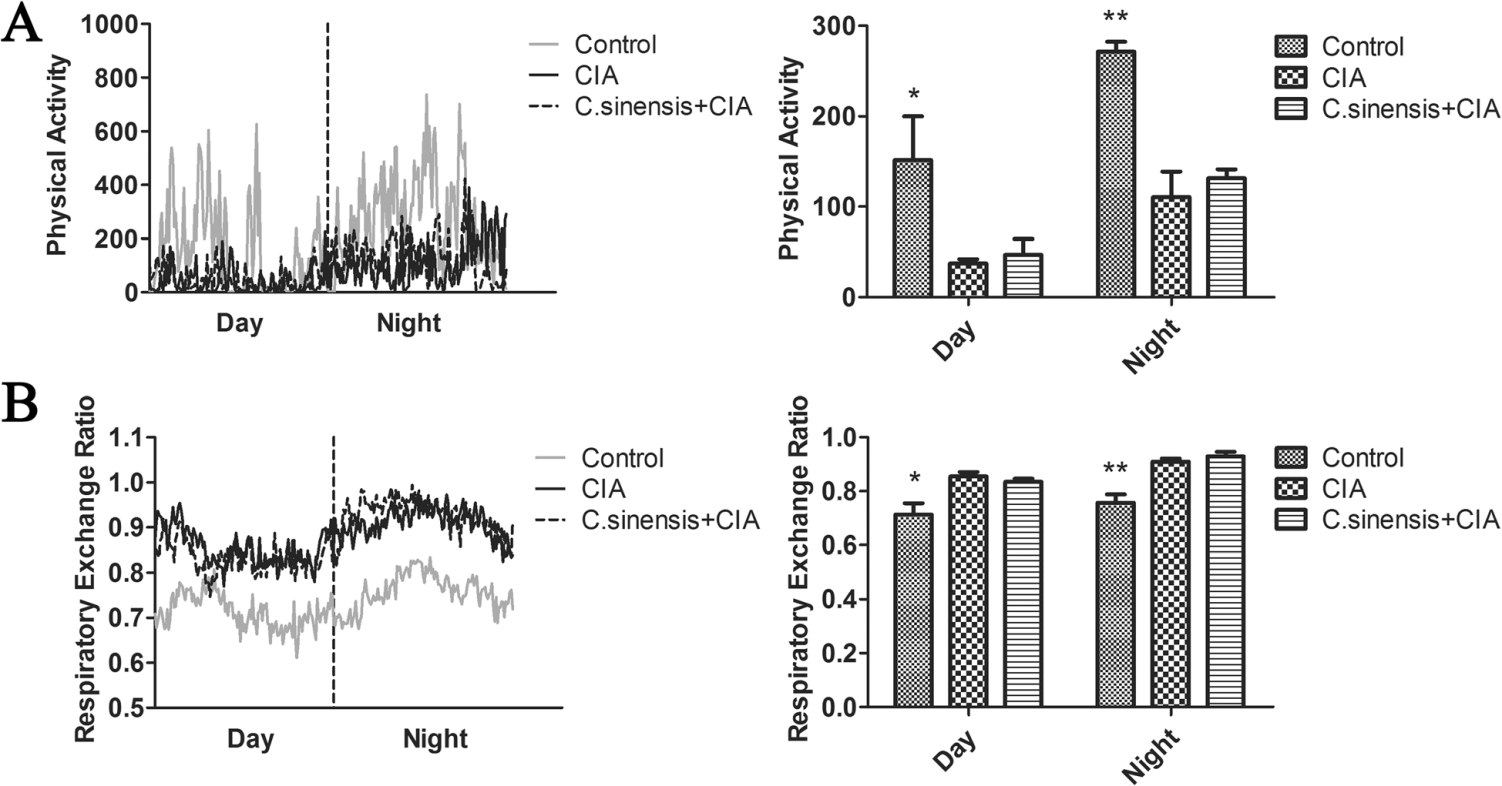
Mice were housed in automated metabolic cages. Physical activity (**a**), respiratory exchange ratio (**b**), were monitored. For bar graphs,data are shown as means+SEM (*n* = 5); for line graphs,data are shown as the mean for each group (*n* = 5) during a 24-h cycle. Asterisks mark statistically significant difference (*P < 0.05,**P < 0.01)

CIA could induce the change of the joint swelling in mice, and the motion of joints could be altered. In the present study, we detected the physical activity of the mice in three groups. The physical activity of the mice in both the *C.sinensis* + CIA group and the CIA group were significantly reduced than those in mice in the control group. However, there was no difference in *C.sinensis* + CIA and CIA group (Fig. 3a). We further analyzed the respiratory exchange ratio (RER) in mice in three groups, and the result showed that the RER of mice in the *C.sinensis* + CIA group and CIA group were higher than those in mice from the control group (Fig. 3b). No differences of RER were found in mice between the *C.sinensis* + CIA group and the CIA group (Fig. 3b).

### *C.sinensis* infection alters the humoral immune response in CIA mice

**Fig. 4:**
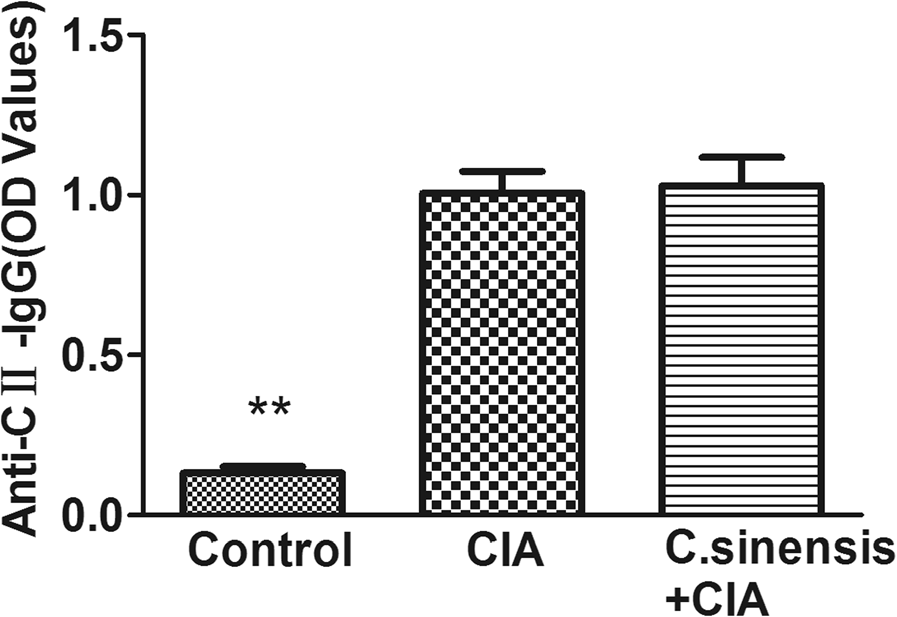
The anti-collagen IgG in the serum were tested by ELISA. Both *C.sinensis* + CIA group and CIA group have higher level of anti-collagen IgG(**P < 0.01). But there was no difference between *C.sinensis* + CIA group and CIA group

To estimate the effect of *C.sinensis* infection on the humoral anti-collagen response in mice, we measured the levels of anti-collagen IgG in the serum in the three groups. As shown in Fig. 3, compared with the mice in the control group, a significantly increased level of anti-collagen IgG was present in mice in both *C.sinensis* + CIA and CIA groups. But there was no difference in mice in the *C.sinensis* + CIA group and CIA group (Fig. 4).

### The changes in immune cells after *C.sinensis* infection in CIA mice

**Fig. 5:**
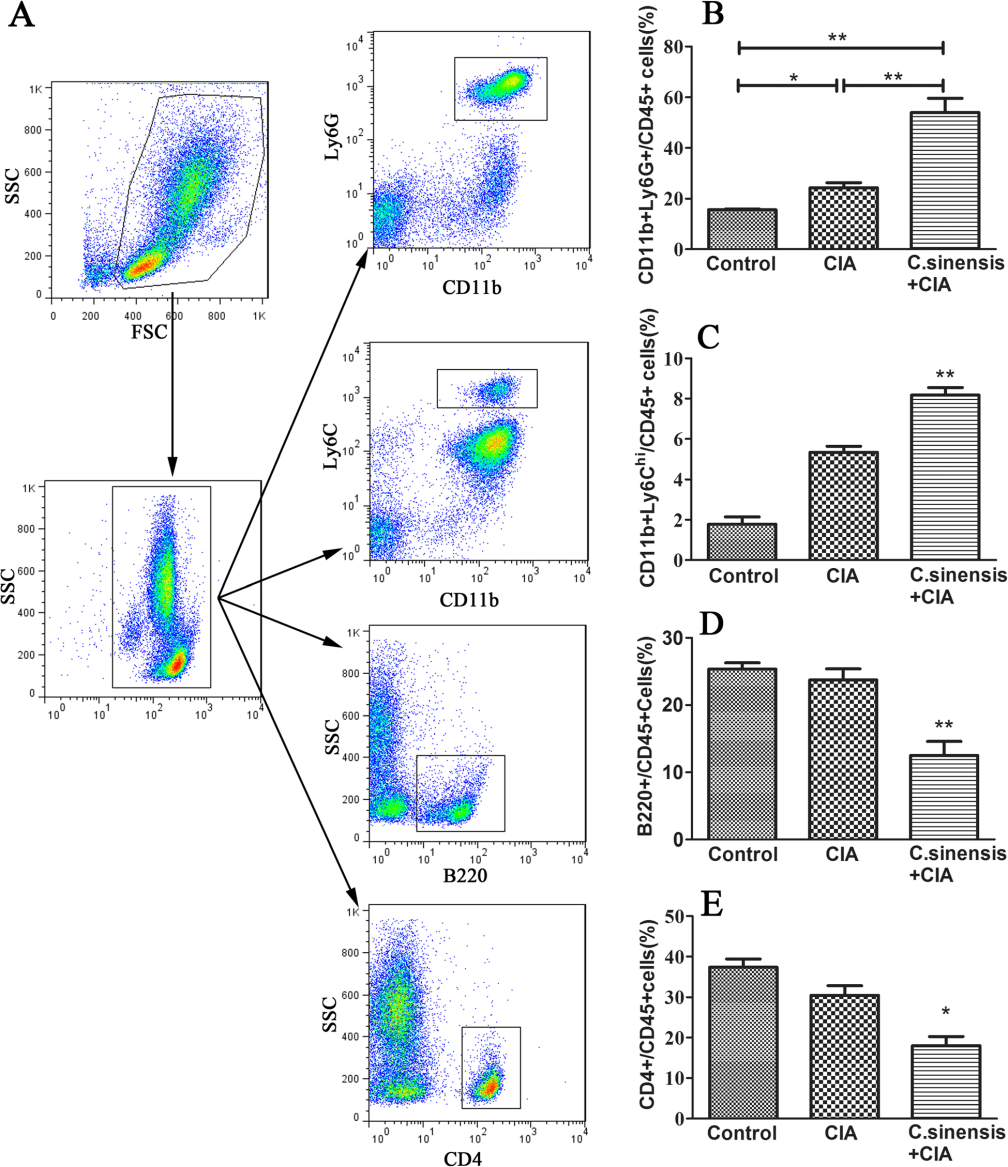
Effects of C.sinensis infection on immune cells. Peripheral blood were collected from mice. **a** Neutrophils, Ly6c + monocytes, B cells and CD4 + T cells were analyzed by flow cytometry. **b**, **c** Compare to CIA group and control group, the percentage of neutrophils and Ly6c^hi^monocytes were higher (**P < 0.01). **d**, **e** However the percentage of B cells and CD4 + T cells were lower than CIA group and control group (*P < 0.05)

Current studies indicated that neutrophils, monocytes, T cells, and B cells, can be attracted and quickly recruited into the sites of infection, and play essential roles in rheumatoid arthritis [11, 12]. In the study, we assessed whether *C.sinensis* infection could affect these immune cells in CIA mice. As the results showed in Fig. 5, the percentages of neutrophils and CD11b^+^Ly6c^hi^monocytes in mice from the *C.sinensis* + CIA group were significantly higher than those in mice in the CIA group and control group. However, compared with the CIA group and control group, the percentages of CD4^+^T cells and B cells were lower in the *C.sinensis* + CIA group.

**Fig. 6:**
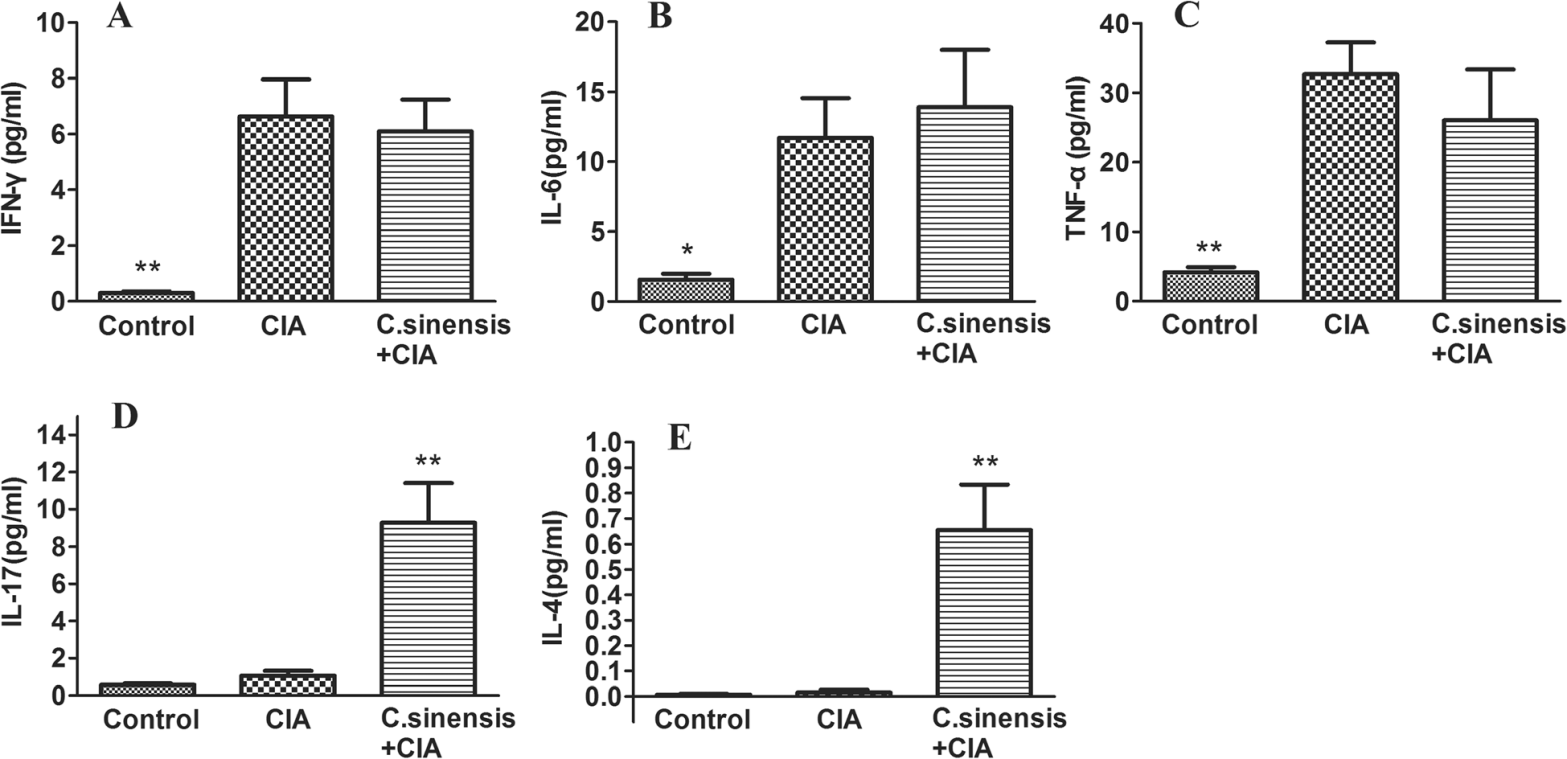
Effects of C.sinensis infection on systemic cytokines. Serum were collected and analyzed for cytokines by CBA as described in materials and methods. **a**, **b**, **c** Data show that both CIA group and *C.sinensis* + CIA group,the level of IFN-γ, IL-6 and TNF-α were higher than control group. But no difference were found in CIA group and *C.sinensis* + CIA group. However, *C.sinensis* infection can alter the level of IL-17 and IL-4. **d**, **e** The expression of IL-17 and IL-4 were significantly higher than CIA group. Asterisks mark statistically significant difference (*P < 0.05, **P < 0.01)

### The changes of cytokines after *C.sinensis* infection in CIA mice

To explore the effect of *C.sinensis* infection on cytokines in the CIA, IL-4, IL-6, IFN-γ, IL-17, and TNF-α in serum in the mice in three groups were detected. As shown in Fig. 6, the levels of serum IL-4 and IL-17 were significantly higher in the *C.sinensis* + CIA group than those in the CIA group and control group. Although compared to control group, higher levels of serum IL-6, IFN-γ and TNF-α were found in *C.sinensis* + CIA group and CIA group, no difference in IL-6, IFN-γ and TNF-α levels was found in *C.sinensis* + CIA group and CIA group.

## Discussion

RA is an inflammatory disorder that mainly affects the joints and synovial tissue and leads to pain and physical disability. The pathogenesis of RA is complex and the etiology is unknown. So far, despite many avenues, including antirheumatic drugs and immune-modulate therapies, have pursued, there are still no effective, safe and affordable treatments for the disease. Previous studies have indicated that certain parasitic infections could reduce the incidence and severity scores of RA [13]. In this study, we evaluated whether *C.sinensis* infection affects RA by using CIA mice models.

To confirm the mice were successfully infected with *C.sinensis*, the livers of mice were obtained for pathological analysis. The infiltration of several inflammatory cells was found in hepatic portal areas. Besides, the destruction of liver tissue structure caused by fibroblast proliferation and collagen deposition, and the destruction of bile duct structure that characterized with cholangiocyte hyperplasia, narrowing of bile duct lumen and periductal fibrosis were found (Fig. 1). These results were consistent with the typical pathological of *C.sinensis* infection as described in our previous research [14].

Current studies show that different parasitic infections have distinct effects on rheumatism. For example, *Schistosoma japonicum* infection could reduce the severity of rheumatic diseases in the CIA mice [7]. However, Graepel et al. reported that when mice infected with *Hymenolepis diminuta*, the arthritis was exaggerated, and the mice had more severe clinical symptoms [15]. We assessed the effect of *C.sinensis* infection on CIA in mice. Obvious joint swelling and significant clinical scores were found in CIA mice in this study. Besides, with pathological analysis, compared to CIA mice, many inflammatory cells infiltration around the joints, fibroblastic proliferation, and pannus formation were found in *C.sinensis* + CIA mice (Fig. 2). Based on the results of disease score and pathological changes, our data indicated that *C.sinensis* infection could increase the severity of the disease.

Collagen is an important component of cartilage. In rheumatoid arthritis, type-II collagen is a critical autoantigen, and it could induce the production of specific antibodies. Besides, the anti-type-II collagen antibodies could form immune complexes and activate complement in RA patients [16, 17]. In the study, we found that the levels of anti-type-II collagen antibody were elevated in CIA mice, while *C.sinensis* infection in mice could not change the levels of anti-type-II collagen antibody in the CIA model.

With joint swelling, pain and the development of arthritis, the physical activities of *C.sinensis* + CIA mice and CIA mice were found to be seriously limited. However, the mice in the CIA group and the *C.sinensis* + CIA group had the same performance. Interestingly, the RER in the CIA group and *C.sinensis* + CIA group were higher than that control group. It was reported that mice with chronic inflammation have increased RER [18], and the mice could alter their metabolism as a response to colonic inflammation. In the study, no difference in RER was observed in *C.sinensis* + CIA mice and CIA mice. These results suggested that the CIA could decrease the physical activities but increase RER, while *C.sinensis* infection has no significant impacts on the physical activities as well as RER in the mice model we built.

Currently, serveral studies indicated that multiple immune cells were involved in the development of arthritis. For example, neutrophil could be enrolled in the site of inflammation and play an important role in the disease [11]. In RA patients, 90% of cells in the synovial fluid are neutrophils [19]. Besides, neutrophils can also be seen in the pannus and cartilage. Citrullinated autoantigens and neutrophil extracellular traps (NETs) can be taken up by fibroblast-like synoviocytes (FLS), and then presented to T cells. The neutrophil is considered as a bridge connecting FLS to T cells to promote the development of the disease [20]. Furthermore, it has been reported that neutrophil depletion can inhibit the progression of arthritis [21, 22]. LY6C^hi^ monocytes is capable of recruiting to the sites of inflammation, and after extravasation, they can differentiate into macrophages and dendritic cells [23, 24]. Besides, after migrated to inflammatory joints, monocytes could contribute to Th17 differentiation, local antigen presentation, and osteoclastogenesis, and make a significant contribution during CIA development. Moreover, clinical and experimental studies indicated that the number of Ly6C^hi^ monocytes is associated with the severity of the disease, and Ly6C^hi^ monocytes depleting can reduce the inflammation and bone erosion in CIA [25, 26]. B cells and T cells also play important roles in RA. On the one hand, in the early stage of arthritis, B cells, served as antigen-presenting cells, could present antigens to CD4 + T cells, and activate CD4^+^T cells to secrete IL-2 and IFN-γ in the synovial membrane [12, 27]. On the other hand, T-B cell interaction also activate B cells to differentiate into plasma cells, and produce antibodies, autoantibodies, and cytokines, to contribute to the pathogenesis of rheumatoid arthritis [28]. Meanwhile, the activation of T and B cells further causes the production of associated cytokines and chemokines and results in the formation of feedback loops between T and B cells [29]. In the study, considering the importance of these immune cells in arthritis; we are interested in detecting whether the *C.sinensis* infection has an impact on immune response mediated by neutrophils, monocytes, T cells and B cells mediated in ICA.

Based on the data as shown in Fig. 5, we found that the number of neutrophils and LY6C^hi^ monocytes in both the CIA group and the *C.sinensis* + CIA group were higher than those in control group. Furthermore, compared to the CIA group, the number of neutrophils and LY6C^hi^ monocytes were higher in the *C.sinensis* + CIA group. Besides, compared with the control group and the CIA group, the number of B cells and CD4^+^T cells in the *C.sinensis* + CIA group were reduced. Taken together, these results suggested that *C.sinensis* infection could cause the dysfunction of immune response mediated by neutrophils, LY6C^hi^ monocytes, CD4^+^T cells, and B cells in the CIA.

Cytokines are usually secreted by immune cells, and contribute to the activation, differentiation, migration, and survival of host cells with various types in vivo. It has been shown that several cytokines, including IL-6, TNF-α, IL-17, and IFN-γ play dominant roles in RA [30]. These cytokines could regulate immune response through complex signal pathways and thus affect the pathological process of RA. For example, IL-6 is a cytokine produced by a variety of cells, including B cells, T cells, and macrophages. High level of IL-6 can be detected in RA patients and participates in joint destruction by acting on neutrophils and pre-osteoclasts, through RANKL dependent or RANKL independent mechanisms [3]. The elevation of TNF-α has also been observed in the serum of RA patients. TNF-α can strongly promote osteoclast differentiation with RANKL, and causes pannus formation and inflammatory bone resorption [31]. Besides, blocking the function of TNF-α and IL-6 by monoclonal antibodies could significantly reduce the pathobiology of RA [32, 33]. IL-17 is mainly produced by Th17 cells and could activate macrophages and synovial fibroblasts, increase the production of MMP1 and MMP3 as well as inflammatory cytokines, including TNF-α, IL-6, and IL-1. In addition, IL-17 can also recruit neutrophils and monocytes to the site of inflammation and promotes osteoclast differentiation, which leads to cartilage destruction and bone erosion in arthritis [34]. IFN-γ is a Th1-type cytokine that facilitates leukocytes transfer through the endothelial layer by promoting the expression of chemokines. IFN-γ also activates macrophages to increase antigen presentation and inducible nitric oxide synthase (iNOS) during the development of arthritis [35, 36]. In the study, we found that, compared to the control group, the cytokines, including IL-6, TNF-α and IFN-γ were elevated in both the CIA group and the *C.sinensis* + CIA group. But there was no difference between CIA group and *C.sinensis* + CIA group. These results indicated that *C.sinensis* infection has no significant effect on the expression of these three kinds of cytokines. In addition, the levels of IL-4 and IL-17 in the *C.sinensis* + CIA group were found to be significantly higher than those in both the CIA group and the control group. During the acute phase of infection, *Clonorchis sinensis* is capable of inducing the host immune response towards a strong Th1 immune response, which could induce the expression of IFN-γ. With the development of infection, Th1 immune response could shift to Th2, following the overexpression of IL-4 [10, 37]. These results suggested that the immune response in the CIA may be influenced by *C.sinensis* infection, via increased expression of IL-4 and IL-17 in mice.

## Conclusions

Taken together, it has been reported that certain parasites could significantly attenuate the clinical signs of arthritis in mice, and the associated mechanisms are related to immune reaction mediated by parasite infection [7, 13]. However, in this study, our results indicated that *C.sinensis* can’t ameliorate the sympt om of arthritis. Furthermore, to some extent, *C.sinensis* can exacerbate an experimental model of arthritis, and the effect of *C.sinensis* infection on arthritis may be associated with the change of immune cells and associated cytokines.

## Methods

### The collection of *C.sinensis* metacercariae

Fresh fish infected with *C.sinensis* metacercariae were collected and transported from Guangxi Autonomous Region, People’s Republic of China. There was no specific permission required during the collection. Then, the fish were minced and digested with artificial gastric juice (0.7% pepsin in 1% HCL) at 37 °C for 12 h, as described by Yan, et al. [37]. The digested mixture was filtrated through 1000 μm, 300 μm and 106 μm sieves. Then the metacercariae were identified and collected with dissecting microscope.

### Animals

BALB/c mice (6-8 week) were purchased from Shanghai Laboratory Animal Co., Ltd. (SLAC, Shanghai, China). All mice were maintained in the SPF laboratory of Xuzhou Medical University. All animal experiments were approved by the Animal Care and Use Committee of Xuzhou Medical University (No. SCXK < SU > 2015–0030).

### Induction and clinical assessment of arthritis

Mice were randomly divided into 3 groups as follows: i) Control group (Control), ii) type II collagen-immunized group (CIA group), iii) *C.sinensis* infection and type II collagen-immunized group (*C.sinensis* + CIA group). In *C.sinensis* + CIA group, the mice were infected about 45 *C.sinensis* metacercariae by intragastric administration. Four weeks later, the mice in ii and iii groups were immunized with 100 μg of bovine type-II collagen (Chondrex, 20,022, USA) in complete Freund’s adjuvant (Sigma, SLBM9312, USA) intradermally on the back of the mice. Two weeks later, the second immunization with 100 μg of type-II collagen in incomplete Freund’s adjuvant (Sigma, SLBM7415, USA) was performed. From 3 weeks post-immunization, the severity of arthritis was evaluated every 3 days. The arthritis score was quantified based on swelling and redness (graded from 0 to 4 for each paw; 0: normal; 1: slight swelling and redness; 2: moderate swelling and redness; 3: severe swelling and redness in large joints and moderate swelling and redness in small joints; 4: most severe swelling and redness in large joints and severe swelling and redness in small joints) [38].

### Joint histology

Mice were sacrificed on day 35 with carbon dioxide. The ankle joints were collected and fixed in 4% paraformaldehyde (PFA) for 1 week. Following fixation, the joints were decalcified in 12.5% EDTA2Na for 1 month, with the solution changed every 2 days. Next, the joint tissues were embedded and sectioned into 4 μm-thick slices. The cartilage destruction, vascular proliferation, inflammatory cell infiltration, and synovial hyperplasia were assessed by hematoxylin-eosin and masson staining.

### Detection of antibodies against type-II collagen

Serum was collected from mice on day 35 after first type-II collagen immunization. The level of anti-type-II collagen IgG was detected by ELISA. In brief, 96-well ELISA plates were coated with type-II collagen at 5 μg/ml overnight. Serum sample was incubated and specific secondary antibodies were used. After the enzymatic reaction, the absorbance was measured at a 450 nm wavelength.

### Ambulatory activity and indirect calorimetry

Physical activity and indirect calorimetry were assessed by Columbus Comprehensive Lab Animal Monitoring System (CLAMS, Columbus, USA). Briefly, mice were transferred into metabolic cages for 1 day of acclimation. Then the physical activity and respiratory exchange ratio (RER) were recorded every 5 or 6 min for 48 h.

### Flow cytometry

The number of neutrophils, B cells, and CD4 + T cells was analyzed by flow cytometry. Briefly, peripheral blood mononuclear cells (PBMCs) from mice in different groups were stained with anti-mouse CD45 (BD Biosciences, 561,037, USA), anti-mouse CD4 (BD Biosciences, 553,651, USA), anti-mouse B220 (ebioscience, 69–0452-82, USA), anti-mouse CD11b (BD Biosciences, 550,993, USA), anti-mouse Ly6G(BD Biosciences, 560,599, USA), anti-mouse Ly6C (ebioscience, 25–5932-80, USA) for 30 min. After washing with PBS, the cells were analyzed with BD FACSCanto II flow cytometer.

### Cytometric bead Array (CBA)

Serum was collected from mice on day 35 after first type-II collagen immunization. The concentrations of IL-4, IL-6, IFN-γ, IL-17, and TNF-α were detected by CBA assay (BD Biosciences, 560,485, USA) with BD FACSCanto II flow cytometer, and the cytometric data were analyzed with FCAP Array software v 3.0 (BD Biosciences).

### Statistical analysis

All data were analyzed by SPSS18.0 and presented as mean ± standard deviation. One-way ANOVA was performed to analyze the statistical significance. *P* < 0.05 was considered statistically significant.

## Data Availability

The data supporting the conclusions of this article are included within the article.

## Abbreviations

C.sinensis: *Clonorchis sinensis*
CBA: Cytometric Bead Array
CIA: Collagen-induced arthritis
ELISA: Enzyme linked immunosorbent assay
RA: Rheumatoid arthritis
RER: Respiratory exchange ratio
